# DNA Double-Strand Break Repair as Determinant of Cellular Radiosensitivity to Killing and Target in Radiation Therapy

**DOI:** 10.3389/fonc.2013.00113

**Published:** 2013-05-10

**Authors:** Emil Mladenov, Simon Magin, Aashish Soni, George Iliakis

**Affiliations:** ^1^Institute of Medical Radiation Biology, University of Duisburg-Essen Medical SchoolEssen, Germany

**Keywords:** DNA double-strand breaks, ionizing radiation, homologous recombination repair, radiosensitization, cancer

## Abstract

Radiation therapy plays an important role in the management of a wide range of cancers. Besides innovations in the physical application of radiation dose, radiation therapy is likely to benefit from novel approaches exploiting differences in radiation response between normal and tumor cells. While ionizing radiation induces a variety of DNA lesions, including base damages and single-strand breaks, the DNA double-strand break (DSB) is widely considered as the lesion responsible not only for the aimed cell killing of tumor cells, but also for the general genomic instability that leads to the development of secondary cancers among normal cells. Homologous recombination repair (HRR), non-homologous end-joining (NHEJ), and alternative NHEJ, operating as a backup, are the major pathways utilized by cells for the processing of DSBs. Therefore, their function represents a major mechanism of radiation resistance in tumor cells. HRR is also required to overcome replication stress – a potent contributor to genomic instability that fuels cancer development. HRR and alternative NHEJ show strong cell-cycle dependency and are likely to benefit from radiation therapy mediated redistribution of tumor cells throughout the cell-cycle. Moreover, the synthetic lethality phenotype documented between HRR deficiency and PARP inhibition has opened new avenues for targeted therapies. These observations make HRR a particularly intriguing target for treatments aiming to improve the efficacy of radiation therapy. Here, we briefly describe the major pathways of DSB repair and review their possible contribution to cancer cell radioresistance. Finally, we discuss promising alternatives for targeting DSB repair to improve radiation therapy and cancer treatment.

## Introduction

During the past few decades extensive efforts have been made to improve cancer therapy both by establishing more successful treatment approaches, as well as by developing effective means for early diagnosis. Despite advances in many fronts, radiation remains one of the most successful treatment modalities for solid cancers that is applied to over 50% of all cancers at one stage of their management (Delaney et al., 2005; Connell and Hellman, 2009; Ahmad et al., 2012; Siegel et al., 2012).

Radiation therapy aims to cure cancer by eradicating tumor cells. The tool of radiation therapy, ionizing radiation (IR), induces a plethora of DNA lesions, including oxidative base damages, single-strand breaks (SSBs), and double-strand breaks (DSBs), which affect the DNA integrity or alter its chemical nature (Ward, 1990). Among these lesions, DSBs have been reported to trigger the most detrimental effects on genome stability, and have been identified as the main contributors to IR induced cell killing through the formation of chromosomal aberrations (Povirk, 2006; Iliakis et al., 2007).

However, while treating tumors, radiation also always reaches normal tissue risking the development of side effects and the generation of secondary malignancies. As a result, the central challenge of radiation therapy is to maximize tumor cell killing and minimize at the same time the normal tissue side effects. Modern conformal, intensity-modulated radiation therapy approximates this goal by optimizing radiation dose deposition between tumor and the surrounding normal tissue. Further improvements are possible using biological approaches that exploit differences in radiation response between normal and tumor cells and aim at protecting normal cells while maximizing the radiation response of tumor cells. Thus, radiosensitizing agents offer a benefit when radiosensitization occurs rather specifically in cancer cells.

The observation that components of the homologous recombination repair (HRR) pathway are aberrantly expressed in many tumors (Connell et al., 2006; Klein, 2008; Miyagawa, 2008; Tennstedt et al., 2012) and the correlation between tumor radioresistance, poor prognosis, and increased HRR activity make this repair pathway an attractive target with potential for differential responses. This view is further reinforced by the highly selective cell killing exerted by PARP-inhibitors in HRR deficient cells. However, similar conclusions have been also drawn for other DSB repair pathways (see below).

In the present review, we give a brief overview of the current state of knowledge in DSB repair and outline how this information may be harnessed to improve radiation therapy.

## The DSB Repair Arsenal

Double-strand breaks are generated randomly in the genomic DNA after exposure of cells to IR, or after treatment with radiomimetic drugs, DNA replication inhibitors, or topoisomerase poisons (Povirk, 2012). DSBs also arise randomly throughout the genome from reactive oxygen species generated as byproducts of the cellular metabolism, as well as from errors during DNA replication, the improper elimination of which may contribute to cancer progression (Vilenchik and Knudson, 2003).

Notably, DSBs are also generated in a programed manner as part of important cellular processes, such as the maturation of lymphoid cells or gametogenesis during meiosis (Keeney et al., 1997; Panizza et al., 2011; Schatz and Swanson, 2011). In both cases specific enzymes are involved in the production of DSBs that are generated under stringent control, mostly at pre-defined locations in the genome.

In cancer therapy, the lethal effects of randomly induced DSBs are exploited to eliminate actively proliferating tumor cells. However, since induction of DSBs by IR appears comparable in normal and tumor cells, specificity of IR-toxicity to cancer cells is likely to rely either on their increased proliferative activity, or on defects in the processing of DSBs. Indeed, tumor cells frequently exhibit defects in various DNA repair pathways, which generate opportunities for enhanced treatment efficacy.

Two mechanistically and genetically distinct pathways contribute to the elimination of DSBs from the genome of higher eukaryotes: non-homologous end-joining (NHEJ), which can be subdivided in DNA-PKcs dependent NHEJ (D-NHEJ) and alternative/backup NHEJ (B-NHEJ) (see below), and HRR.

### DSB repair by D-NHEJ

D-NHEJ catalyzes a simple rejoining reaction between two DNA ends irrespective of their origin (Lieber, 2010) and does not require homology at the ends or elsewhere; these facts render NHEJ operational throughout the cell-cycle. Indeed, D-NHEJ is active in all phases of the cell-cycle, where it removes DSBs from the genome with similar efficiency, but possesses only limited functionality for single-ended DSBs that arise during replication (Metzger and Iliakis, 1991; Rothkamm et al., 2003; Helleday et al., 2007).

**Figure 1:**
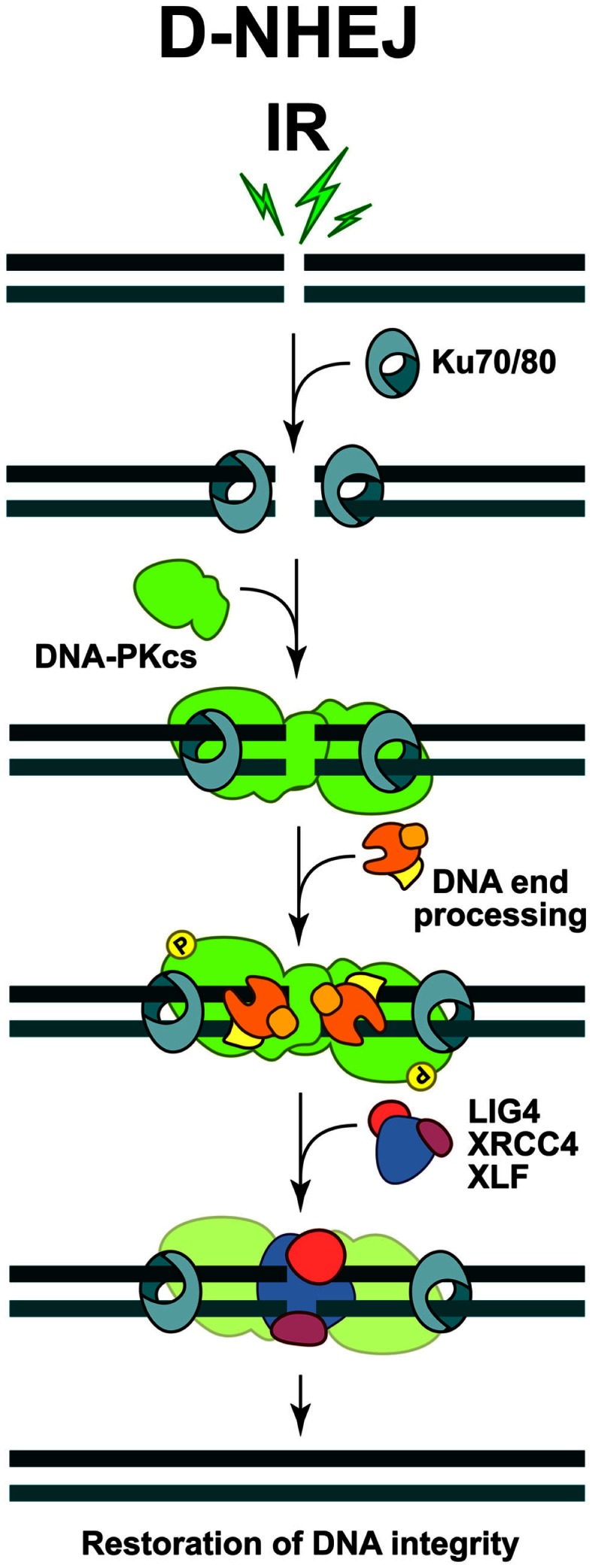
**Schematic representations of DSB repair by non-homologous end-joining**. The process is initiated by the binding of KU heterodimer to the DNA ends, which then recruits DNA-PKcs to form an active DNA-PK holoenzyme. DNA-PK activation helps the recruitment of multiple proteins involved in the limited DNA end-processing (Artemis, pol μ, pol λ, and TDK) required to generate ligatable DNA ends. Ligation is mediated exclusively by the LIG4/XRCC4 complex and is assisted by the ligation mediator XLF. At the end of this process the DNA integrity at the break is restored, but the DNA sequence at the junction may be altered.

The key steps of the classical form of NHEJ are summarized in Figure 1. The high affinity of KU heterodimer for free DNA ends (1–10 × 10^−9^ M, depending on the DNA end-structures), makes it the ultimate initiation factor of this repair pathway (Arosio et al., 2002). Indeed, it has been shown that the two subunits of the KU heterodimer, KU70 and KU80, which form an asymmetric toroid structure, are perfectly designed to bind and threat on free DNA ends (Figure 1). The binding of KU to DSBs blocks nucleolytic processing of DNA ends, which is required for the initiation of other DSB repair pathways (see below). However, despite its reported lyase activity (Roberts et al., 2010; Strande et al., 2012), the essential role of KU during NHEJ is to recruit the catalytic subunit of the DNA-PKcs, which dominates and drives the repair of DSBs in cells of higher eukaryotes. Hence, this form of repair has also been termed D-NHEJ (Mladenov and Iliakis, 2011).

The binding and dimerization of DNA-PKcs immobilizes the two DNA ends and thus facilitates the rejoining reaction (Meek et al., 2004). The interactions of DNA-PKcs with KU, as well as the binding of DNA-PKcs to the DNA result in almost 10-fold increase in DNA-PKcs kinase activity. Accumulating evidence shows that a variety of proteins specifically involved in D-NHEJ, or generally in the DNA damage response (DDR), are phosphorylation targets of DNA-PKcs. However, interference with these phosphorylations is often without effect in DSB repair, and the only modification, which severely affects the end-joining efficiency, is elimination of DNA-PKcs auto-phosphorylation (Meek et al., 2004). Depending on the nature of DNA lesions, DNA-PKcs can be phosphorylated at multiple residues, which is a prerequisite for its dissociation from the damaged sites and the recruitment of other repair factors (Figure 1).

Since DSBs generated by IR bear damaged nucleotides at their ends, a limited end-processing by nucleolytic enzymes or DNA polymerases (pol μ and pol λ) is required to generate ligatable ends (Weterings and Chen, 2008) (Figure 1). As a result, sequence changes at the junctions generated by NHEJ are possible and therefore mutations likely. Furthermore, since D-NHEJ rejoins DNA ends indiscriminately, it can lead to translocations and other chromosomal rearrangements that are hallmarks of genomic instability. It is therefore quite surprising that despite such limitations, cells of higher eukaryotes extensively utilize D-NHEJ to remove DSBs from their genome.

The final step during D-NHEJ is mediated by a highly specialized ligation complex consisting of DNA Ligase 4 (LIG4) and the X-ray cross complementing 4 (XRCC4) protein (LIG4/XRCC4) (Figure 1). Assisted by the auxiliary factor XLF (Cernunos), LIG4/XRCC4 mediates ligation that results in fast and efficient restoration of DNA integrity, albeit often at the cost of sequence information loss.

### DSB repair by B-NHEJ

During the past decade a second pathway for rejoining of broken DNA molecules on the basis of NHEJ was discovered and is presently intensively investigated. As with D-NHEJ this repair pathway also lacks means to restore sequence information at the DSB, and as we will discuss later, it also has a higher probability to join unrelated DNA ends.

Initially, analyses of DSB repair using pulse-field gel electrophoresis (PFGE) in cells deficient in components of D-NHEJ, revealed a robust repair activity that was unrelated to HRR (see below) and reflected a different form of DNA end-joining instead (DiBiase et al., 2000; Singh et al., 2009). This alternative form of DSB repair efficiently substituted for D-NHEJ, but appeared to have backup functions, coming to the fore mainly after failure of D-NHEJ; therefore the term B-NHEJ was proposed for this repair pathway (Iliakis, 2009; Mladenov and Iliakis, 2011). Failures of D-NHEJ, which allow function of B-NHEJ, can also occur locally at a specific DSB, even in repair proficient cells, or globally in cells with mutations in genes encoding for D-NHEJ factors, or after treatment with DNA-PKcs inhibitors. Subsequent work documented the function of such alternative pathways of NHEJ in several processes involving the formation of DSBs, such as V(D)J recombination and class switch recombination (Corneo et al., 2007), and were also implicated in cancer formation (Simsek et al., 2011).

Several enzymatic activities have been implicated in this repair pathway, which is now considered to be distinct from D-NHEJ and which may even be further subdivided into sub-pathways (Wang et al., 2005; Rosidi et al., 2008; Zha et al., 2009; Lee-Theilen et al., 2011; Mladenov and Iliakis, 2011).

**Figure 2:**
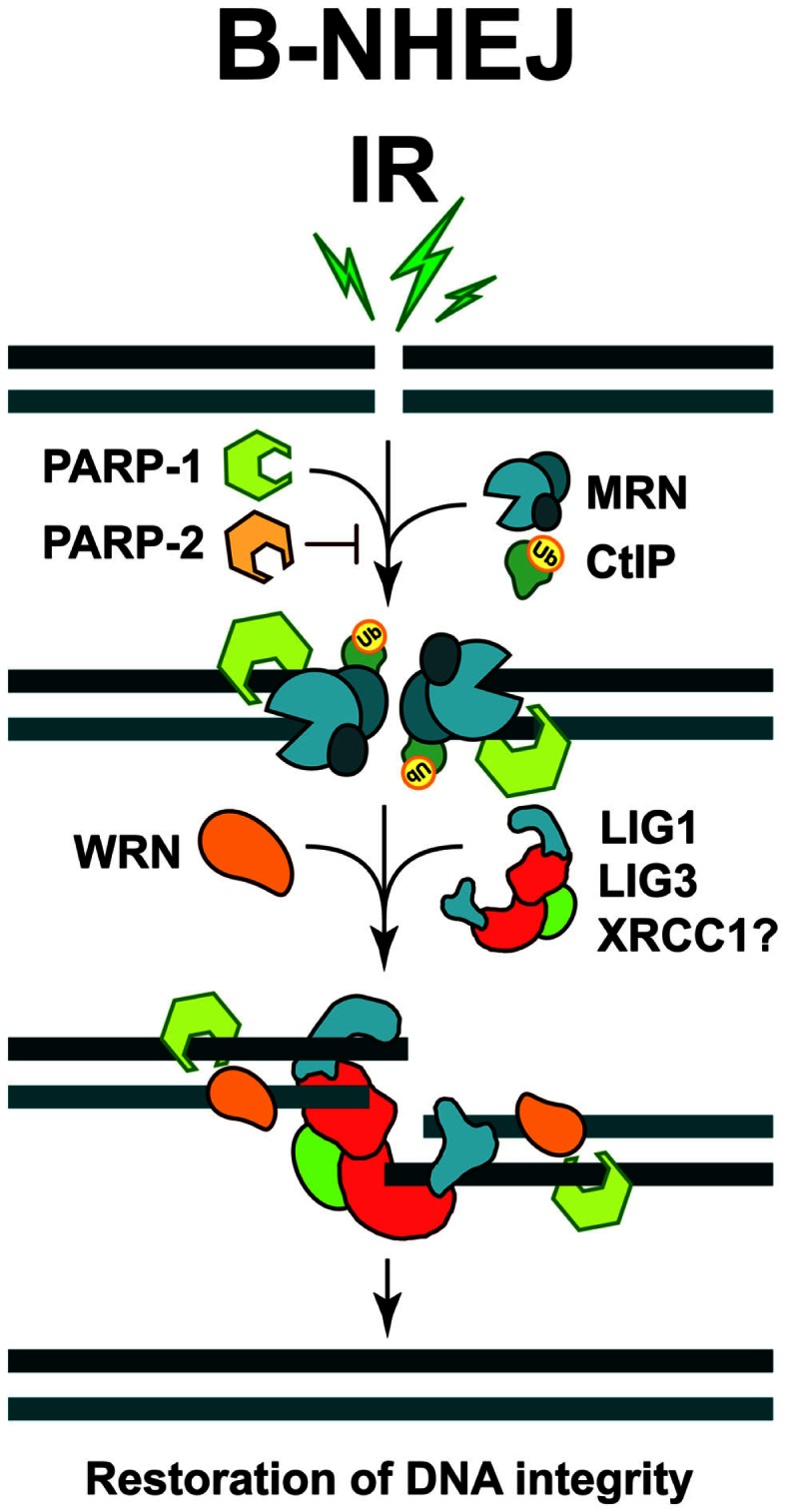
**Repair of DSBs by B-NHEJ**. This pathway remains incompletely characterized. Factors implicated include PARP-1, the MRN complex, CtIP, and WRN. Ligation is mediated by LIG3 or LIG1. The restoration of DNA integrity through this repair pathway is also associated with sequence information loss at the junction, and more importantly, by increased risk of joining unrelated DNA ends to generate translocations and other genomic rearrangements.

A major protein implicated in B-NHEJ is poly (ADP-ribose) polymerase 1 (PARP-1), which plays a main role in the repair of SSBs (see below) and which may effectively compete with KU heterodimer for DNA end-binding (Wang et al., 2006). It has been reported that PARP-1 facilitates the repair of DSBs by B-NHEJ, while another member of the PARP family, PARP-2 strongly suppresses it (Robert et al., 2009) (Figure 2). It has also been reported that B-NHEJ benefits from microhomology at the break sites, which may be best found if the DNA ends become resected. Indeed, MRE11 and C-terminal binding protein 1 interacting protein (CtIP), both involved in DNA end-resection during HRR (see below), were found to facilitate B-NHEJ (Zha et al., 2009; Lee-Theilen et al., 2011) (Figure 2). However, it is important to point out that B-NHEJ does not exhibit a strict requirement for microhomology, therefore, this repair pathway should not be considered as a microhomology dependent (Mansour et al., 2010).

Backup NHEJ, like D-NHEJ, is active in all phases of the cell-cycle, but its activity is significantly potentiated during S and G2, probably due to the increased activity of DNA end-resection enzymes in these cell-cycle phases. Therefore, it is likely that B-NHEJ also operates as a backup to HRR in the G2 and S-phases of the cell-cycle (see below). Notably, B-NHEJ is severely compromised when D-NHEJ deficient cells enter a plateau phase of growth or are deprived of serum (Singh et al., 2011, 2012). An intriguing and still unexplained observation is that this effect is not observed in DNA-PKcs deficient cells (Singh et al., 2011).

A ligation activity finalizing B-NHEJ is DNA Ligase 3 (LIG3), a versatile ligase, which in complex with XRCC1 also participates in the repair of SSBs and DNA base damages (Wang et al., 2005; Della-Maria et al., 2011) (Figure 2). Assisted by its unique structural properties, LIG3 ensures the ligation of both DNA strands during DSB repair (Ellenberger and Tomkinson, 2008). However, despite reports to the opposite (Audebert et al., 2004), the role of XRCC1 in LIG3 function during B-NHEJ remains unclear (Della-Maria et al., 2011; Boboila et al., 2012). Recent evidence also implicates DNA Ligase 1 (LIG1) in B-NHEJ (Simsek et al., 2011; Paul et al., 2013). Thus, it appears that while LIG4 is specifically dedicated to D-NHEJ, LIG1, and LIG3 can efficiently support B-NHEJ.

Furthermore, interesting regulatory proteins were implicated in B-NHEJ. The Werner syndrome helicase (WRN), together with LIG3, was found upregulated in chronic myelogenous leukemia (CML), where several D-NHEJ activities are suppressed. Under these conditions WRN and LIG3 form a stable complex, which is recruited to DSBs, thus activating the ligation process (Sallmyr et al., 2008) (Figure 2). Moreover, a form of error-prone repair, with characteristics of single-strand annealing (SSA), was described in many myeloproliferative disorders, which are characterized by the formation of oncogenic fusion tyrosine kinases, including BCR/ABL, TEL/ABL, TEL/JAK2, and TEL/PDGFBR (Cramer et al., 2008). This form of repair contributes to the accumulation of intrachromosomal deletions and translocations, a hallmark of the B-NHEJ repair pathway; therefore it has been suggested that it might be a sub-pathway of alternative DSB repair mechanisms (Mladenov and Iliakis, 2011).

Another factor implicated in B-NHEJ is histone H1, which enhances the rejoining activity of LIG3 presumably by facilitating the synapsing of DNA molecules (Rosidi et al., 2008).

Backup NHEJ is much slower than D-NHEJ and is highly error-prone causing translocations and other genomic rearrangements with high probability. Moreover, a high number of B-NHEJ associated genetic rearrangements have been observed in chromosomal translocations associated with both spontaneous and therapy-related cancers (Greaves and Wiemels, 2003). Thus, B-NHEJ-derived mutations appear to be associated with cancer development and may support tumor progression – particularly when classical NHEJ or HRR are compromised (Bennardo et al., 2008). It is therefore conceivable that activation of B-NHEJ fuels the evolution of cancer, and that it might also serve as target in specialized cancer therapies.

### DSB repair by HRR

**Figure 3:**
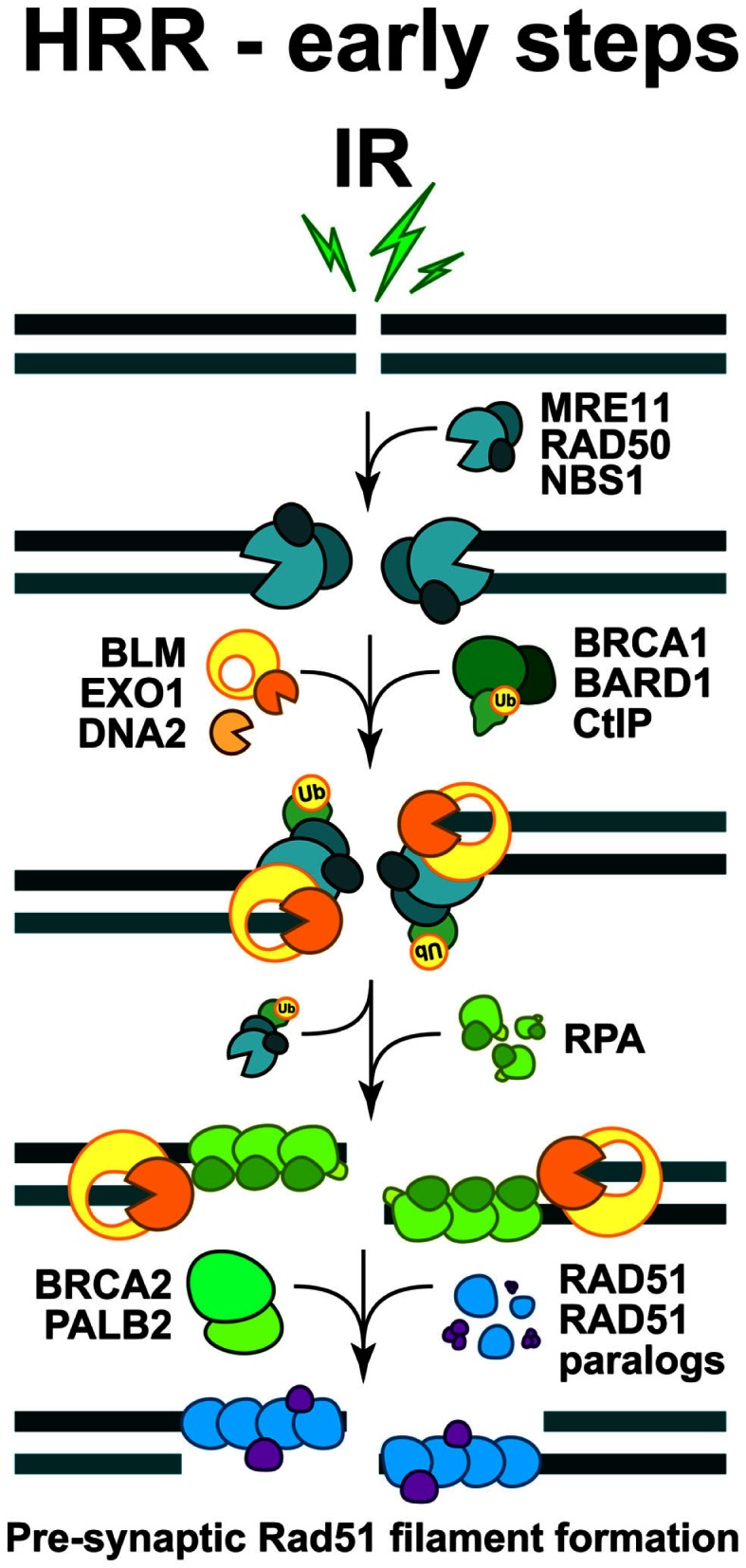
**Schematic overview of the early steps of HRR**. The formation of pre-synaptic Rad51 nucleoprotein filament. The initiation steps and the full sequence of events contributing to the faithful restoration of DNA sequence at the DSB are explained in the text.

**Figure 4:**
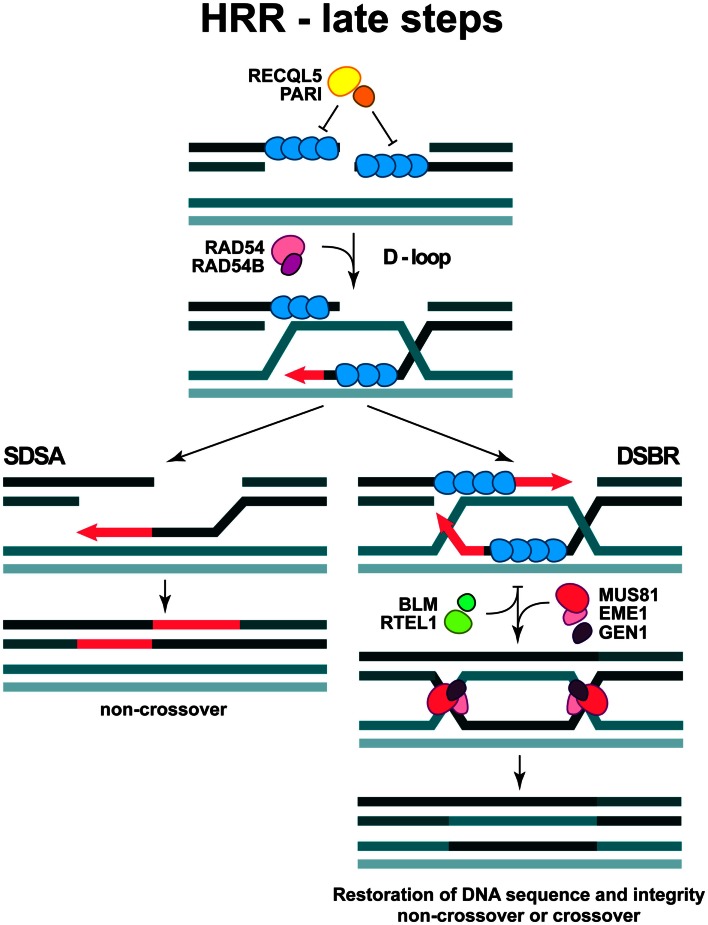
**Late steps in DSB repair by HRR**. The formation of D-loop structure and two sub-pathways are depicted (see text for more details).

The second approach to DSB repair, HRR, requires intact homologous DNA sequences to remove DSBs and to faithfully restore the DNA sequence in their vicinity (San Filippo et al., 2008). One form of HRR (described in Figures 3 and 4) utilizes the sister chromatid as a donor for homologous sequence and is therefore active only in S and G2 phases of the cell-cycle (Onn et al., 2008; San Filippo et al., 2008). In principle, HRR could also be carried out in diploid cells during the G1 phase of the cell-cycle using the homologous chromosome as template. However, the distinct compartmentalization of the nuclear domains of homologous chromosomes make required interactions unfavorable; in fact, it is thought that HRR is actively suppressed in G1 cells in an effort to prevent loss of heterozygosity (LOH) (Paques and Haber, 1999; Aylon and Kupiec, 2004).

DNA end-resection is a necessary requirement for the initiation of HRR, as a long single-stranded 3′-DNA overhang has to be formed in order to start homology search (West, 2003). Activities implicated in diverse aspects of end-resection include the MRE11-RAD50-NBS1 (MRN complex), the CtIP, as well as Exonuclease 1 (EXO1), DNA2, and the Bloom’s syndrome helicase (BLM) (see below) (Figure 3). In order to execute their function during HRR initiation, the MRN complex is quickly recruited to DSB, where it cooperates with CtIP to promote end-resection (Sartori et al., 2007; Kousholt et al., 2012; Leslie, 2013). It is thought that end-resection defines the point of no-return in the decision to process a DSB by HRR. Therefore, the formation of single-stranded DNA regions is frequently used as a surrogate for ongoing HRR. However, as noted above, it is also possible that HRR abrogation after resection will shunt DSBs to B-NHEJ (we will return to this point below).

The combined action of DNA end-resection enzymes results in the formation of single-stranded DNA, decorated by the replication protein A (RPA) (Figure 3). RPA is a heterotrimeric complex, comprising of RPA70, RPA32, and RPA14, which exhibits high affinity for binding to ssDNA regions, such as those formed during DNA replication and occasionally during repair. In the subsequent steps of HRR, RPA is replaced by RAD51 recombinase, which forms a right-handed pre-synaptic RAD51 nucleoprotein filament on the DNA (Figure 3). The replacement of RPA by RAD51 requires the activity of mediator proteins, such as Breast cancer susceptibility gene 2 (BRCA2) and a group of five RAD51 paralogs (RAD51B, RAD51C, RAD51D, XRCC2, and XRCC3), which share 20–30% sequence similarity with the RAD51 recombinase (West, 2003).

The exact function of the RAD51 paralogs remains elusive, but there is evidence that they may form distinct complexes, which facilitate the formation and stabilization of the RAD51 filament (Masson et al., 2001; Forget et al., 2004; Suwaki et al., 2011); they also exhibit some specific functions in telomere maintenance and probably during DDR (Tarsounas et al., 2004; Badie et al., 2009). The accumulation of RAD51 monomers at the damaged sites and the formation of a RAD51 nucleoprotein filament, can be visualized by immunofluorescence microscopy as distinct sub-nuclear RAD51 foci, which are routinely utilized to investigate DSB repair by HRR (Haaf et al., 1995).

The formation of RAD51 nucleoprotein filament is regulated by specific enzymatic activities, which displace RAD51 molecules or facilitate their association. The newly characterized PARI protein together with a RECQL5 helicase may act as a negative regulator of RAD51 filament formation (Karpenshif and Bernstein, 2012) (Figure 4). Moreover, the “anti-recombinase” proteins BLM and RTEL1, could also have negative impact on the already formed RAD51 filament, thus facilitating its dissociation when HRR is disadvantageous (Karpenshif and Bernstein, 2012) (Figure 4).

In the subsequent steps of HRR, the RAD51 nucleoprotein filament invades the intact double-stranded DNA molecule to search for homologous sequences and form a structure termed displacement loop (D-loop). When homology is found (synapsis) DNA synthesis will start elongating the 3′-end of the invading strand (Figure 4). For elongation to commence, RAD51 in the synaptic complex has to be removed from the very 3′ tip of the invading strand to reveal the 3′-OH group for priming; this reaction is facilitated by RAD54 and its interacting partner RAD54B (Li and Heyer, 2009). HRR can take several different routes from this point. Frequently, elongation of the invading 3′-end can continue over a limited distance, followed by displacement of the newly synthesized stretch and re-ligation with the original DNA end resulting in the repair of the DSB (synthesis-dependent strand annealing; SDSA) (Figure 4). This is the most frequent event during DSB repair in cells of higher eukaryotes and is equivalent to gene conversion. Alternatively, second end-capture can occur, leading to the formation of a double Holliday junction (dHJ) (DSB repair; DSBR) (Figure 4). Depending on the resolution of the dHJ by specialized resolving enzymes, GEN1 and possibly MUS81/EME1, this branch of HRR will result in either crossover or non-crossover (gene conversion) outcomes (Constantinou et al., 2002; Wu and Hickson, 2003; Ip et al., 2008) (Figure 4).

First reported in yeast and later in higher eukaryotes, recombination events between areas of homology present in the same DNA molecule could be observed. This process is known as SSA (Ivanov et al., 1996). When this pathway is used to repair a DSB it leads to loss of the DNA segment between the regions of homology and therefore it is considered as mutagenic. The role of SSA in the repair of randomly induced DSBs, such as those generated by IR, remains uncharacterized and is likely to be small. However, there is evidence for a correlation between increased formation of chromosomal aberrations and SSA in cells of myeloproliferative disorders expressing oncogenic fusion tyrosine kinases (Cramer et al., 2008).

Finally, in yeast another mode of HRR – termed break induced replication (BIR) – has been described, that steps into action when one sided DSBs are formed. It is characterized by the initiation of replication through the formation of a replication fork that replicates the entire chromosome past the DSB (Fan et al., 2004). As a result of this peculiarity, BIR can cause extensive LOH (Llorente et al., 2008). However in mammalian cells the action of BIR remains to be demonstrated.

## Regulation of HRR – Ways to Modulate the Repair Pathway Choice

Some competition between the DSB repair processes described above is often considered likely (Sonoda et al., 2006; Shrivastav et al., 2008). Although the basis of DSB repair pathway choice in cells of higher eukaryotes remains largely elusive, it is clear that one important determinant is the position of the cell in the cell-cycle – particularly for HRR (see above). In addition to its requirement for a sister chromatid, HRR is also regulated throughout the cell-cycle in at least two ways: (1) Cell-cycle dependent regulation of the expression levels of proteins involved in HRR and (2) through cyclin dependent kinase (CDK)-dependent phosphorylation of some of its components.

Expression levels of RAD51, Breast cancer susceptibility gene 1 (BRCA1), BRCA2, BLM, and CtIP are all regulated throughout the cell-cycle (Flygare et al., 1996; Yamamoto et al., 1996; Wang et al., 1997; Dutertre et al., 2000; Yu and Chen, 2004; Shrivastav et al., 2008). The respective transcripts and/or proteins are present at low levels in G1 and are hardly detectable or completely absent in non-dividing, G0 cells. Their expression begins with the start of DNA replication and increases further with the progression of cells through S.

**Figure 5:**
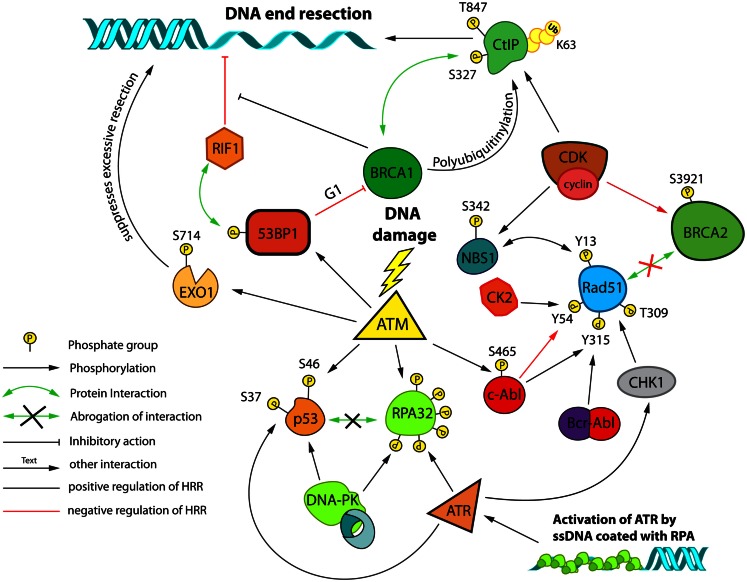
**Post-translational modifications and interactions possibly involved in DSB repair pathway choice and HRR modulation**. A network of proteins controls HRR and is regulated by cell-cycle dependent post-translational modifications. Cell-cycle regulation primarily relies on Cyclin/CDK dependent phosphorylation events. Regulation in response to DNA damage primarily relies on the activities of ATM, ATR, and DNA-PKcs. Control of end-resection plays a central role in this regulation. Resected DNA decorated with heterotrimeric RPA complexes forms a platform for activation of ATR. The schematic focuses on interactions/modifications implicated in the regulation of HRR.

One example of CDK-mediated regulation of HRR is the phosphorylation of Ser-3291 of BRCA2, which counteracts the interaction with RAD51 and thereby negatively regulates HRR activity (Esashi et al., 2005) (Figure 5). Another regulatory CDK-mediated phosphorylation occurs at Ser-432 of NBS1 (Figure 5), which is believed to act as a primary sensor of DSBs. Phosphorylation of Ser-432 on NBS1 stimulates the conversion of DSBs into substrates for HRR in a MRN-dependent manner (Falck et al., 2012).

Although MRE11, another member of the MRN complex, can act as a nuclease and is involved in facilitating resection of ends at sites of DSBs, the formation of extensive ssDNA regions seems to be carried out by other nucleases like EXO1 and possibly DNA2 (Bolderson et al., 2010; Eid et al., 2010; Grabarz et al., 2012; Tomimatsu et al., 2012) (Figure 3) and is facilitated by CtIP and RECQ family members like BLM (Gravel et al., 2008; Mimitou and Symington, 2008; Huertas, 2010).

CtIP not only interacts with the MRN complex, but also with BRCA1 and this interaction is promoted by CDK dependent phosphorylation of CtIP on Ser-327 in S/G2, providing yet another example for cell-cycle regulation of HRR (Yu and Chen, 2004) (Figure 5). CtIP is also polyubiquitinylated in a BRCA1 dependent manner, without being targeted for proteasomal degradation (Yu et al., 2006). However, these are not the only cell-cycle dependent, regulatory modifications CtIP is subjected to. Phosphorylation of CtIP on Thr-847 by CDKs is required for efficient end-resection, and non-phosphorylatable mutants of this site are defective in end-resection, while phospho-mimicking mutants show resection even in the absence of CDK activity (Huertas and Jackson, 2009).

Moreover, it has been previously reported that CtIP is phosphorylated at Ser-664/745 by the Ataxia telangiectasia mutated (ATM) protein kinase, which plays an important role during DSB repair by homologous recombination (Li et al., 2000). Recently, this observation was extended using Ser-664/745-Ala (phosphomutant) and Ser-664/745-Glu (phospho-mimicking) forms of CtIP fused to GFP; it was thus found that ATM is directly engaged in DNA end-resection by activating CtIP via phosphorylation at Ser-664/745 (Shibata et al., 2011).

Although various models emerge as to how HRR is regulated throughout the cell-cycle, the question of choice for a particular repair pathway for each DSB remains poorly understood. There is however, evidence for crosstalk between HRR and NHEJ and it has been reported that DNA-PKcs, ATM, and ATM and Rad3 related (ATR) (in a CHK1/2 independent manner) collaborate to dissociate a p53/RPA-complex by phosphorylating both of its components (Serrano et al., 2012) (Figure 5). Notably, abrogation of these phosphorylations impairs HRR.

A large number of proteins are subjected to post-translational modifications (PTMs) that are elicited by DNA damage or replication stress. We will focus on a small selection of PTMs of proteins that either play a central role in HRR, or exert regulatory functions at key HRR steps.

As mentioned above, CtIP is critical in the regulation of end-resection and is itself post-translationally modified in several ways. Also EXO1, one of the major nucleases implicated in DNA end-resection, has been shown to be phosphorylated at multiple sites. Phosphorylation of EXO1 at Ser-714 by ATM, for example, is required for the recruitment of RAD51 to sites of DSBs (Matsuoka et al., 2007; Bolderson et al., 2010) (Figure 5). Interestingly, this phosphorylation seems to attenuate the nuclease activity of EXO1, suggesting that it might protect from over-resection.

Another level of regulation of end-resection involves p53 binding protein 1 (53BP1) and BRCA1. Interestingly loss of 53BP1 on top of BRCA1 deficiency restores a resistant phenotype in these radiosensitive cells (“synthetic viability”) (Bouwman et al., 2010; Bunting et al., 2010). This has led to a model in which 53BP1 inhibits initiation of DNA end-resection and thus favors the repair by NHEJ. This inhibition is regulated in a negative way by BRCA1 and thus, loss of BRCA1 results in persistent inhibition of end-resection, which can be abolished by removal of 53BP1 (Aly and Ganesan, 2011). A number of very recent publications have now implicated the protein RIF1 as the major downstream effector of the end-protecting function of 53BP1 (Chapman et al., 2013; Di Virgilio et al., 2013; Escribano-Diaz et al., 2013; Zimmermann et al., 2013). One of these studies also found a cell-cycle dependent inhibitory function of 53BP1 on BRCA1 accumulation at DSB in G1 (Escribano-Diaz et al., 2013), which could account for HRR suppression in G1.

The ssDNA generated during resection is immediately covered with RPA. The middle subunit of RPA (RPA32/RPA2) is a target for multiple PTMs, which may have important roles in the subsequent steps of HRR (Figure 5). It has been shown that hyperphosphorylation of RPA32 is required for RAD51 recruitment in response to replication stress induced by hydroxyurea (HU); however such dependencies were not identified after IR or endonuclease induced DSBs (Shi et al., 2010).

Phosphorylations are not the only form of PTMs induced by DNA damage. Besides the well-known waves of ubiquitylation mediated by the E3-ubiquitin-ligases RNF8 and RNF168, modification with small ubiquitin like modifiers (SUMO) also occurs in response to DNA breaks. RPA70 is sumoylated on at least two sites *in vivo*, but this modification is suppressed by a constitutive interaction of the SUMO-specific protease SENP6 (Dou et al., 2010). In response to replication associated DSBs this interaction is abrogated, which allows sumoylation of RPA70 that promotes HRR (Dou et al., 2010). It has also been reported, that the ubiquitin-ligase activity of BRCA1 depends on its sumoylation (Morris et al., 2009). For the BLM helicase multiple sites of sumoylation have been documented as well and shown to influence its nuclear localization and possibly to act as switches shifting BLM-activity between pro- and anti-recombinogenic functions (Eladad et al., 2005; Ouyang et al., 2009).

Upon formation of a RAD51 coated ssDNA the stability of this nucleoprotein filament is critical for the execution of HRR. In mammalian cells helicases like BLM or RECQ5 have been shown to dismantle RAD51 nucleoprotein filaments (Bugreev et al., 2007; Hu et al., 2007) (Figure 4). Very recently it was shown that the helicase domain containing protein PARI suppresses unscheduled and inappropriate HRR at replication forks in human and chicken cells (Moldovan et al., 2012) (Figure 4). This function is mediated by its interaction with PCNA and might be analogous to the function of Srs2 in yeast.

RAD51 itself can also be post-translationally modified in several ways. Following replication stress, RAD51 is phosphorylated in a CHK1 dependent manner on Thr-309 (Sorensen et al., 2005), and also by the c-ABL tyrosine kinase on Tyr-315, which stabilize association of RAD51 with chromatin (Shimizu et al., 2009) (Figure 5). Two additional phosphorylations of RAD51 that occur in a cell-cycle and DNA damage dependent manner have been recently reported: RAD51 is phosphorylated on Ser-14 by PLK1 which licenses phosphorylation of Thr-13 by CK2 (Yata et al., 2012) (Figure 5). Phosphorylation of Thr-13 leads to direct binding of RAD51 to NBS1, which facilitates its recruitment to sites of DNA damage (Yata et al., 2012).

It is evident from the outline above that the cell-cycle regulation of HRR has different levels and is likely to be complex. Identification of these modifications and characterization of their functional significance is likely to have important implications to our understanding of repair pathway choice.

## Regulation of HRR by Tyrosine Kinase Signaling

Besides the above described intrinsic cell-cycle dependent regulatory processes, there is evidence for additional regulatory levels modulating repair of DSBs. An important example is the BCR-ABL fusion tyrosine kinase, a hallmark of CML (Skorski, 2012). The non-mutated c-ABL protein is activated by DNA-PKcs and ATM in response to genotoxic stress (Kharbanda et al., 1995; Baskaran et al., 1997). There are reports suggesting that the constitutively active fusion protein contributes to drug resistance, stimulates HRR, and promotes ectopic recombination events, through either up-regulation of RAD51 levels and/or direct phosphorylation of RAD51 on Tyr-315 (Slupianek et al., 2001, 2002, 2011; Skorski, 2002; Nowicki et al., 2004) (Figure 5).

Up-regulation of mutagenic SSA (see above) by BCR-ABL has also been reported, and BCR-ABL stimulated expression of CtIP and increased DNA end-resection have been suggested as a mechanism (Cramer et al., 2008; Fernandes et al., 2009; Salles et al., 2011). Notably, HRR is negatively regulated by the normal form of c-ABL in irradiated cells by a phosphorylation of RAD51 on Tyr-54 (Yuan et al., 1998) (Figure 5). Another study shows that Rad51 is phosphorylated on both Tyr-315 and Tyr-54 by c-ABL and that these phosphorylations are sequential, with the phosphorylation of Tyr-315 stimulating phosphorylation of Tyr-54 (Popova et al., 2009). Thus, c-ABL may fine-tune recombination repair by balancing activating and inhibitory RAD51 phosphorylations, whereas the constitutively active BCR-ABL protein may cause its hyperactivation with mutagenic consequences. Interestingly, it has been reported, that in ABL-positive CML cells, there is also a shift toward more mutagenic NHEJ repair processes. Sallmyr et al. found LIG4 and Artemis to be down-regulated in these cells, while LIG3 was upregulated. Since in these cells error-prone NHEJ was observed, the authors concluded that the activity of B-NHEJ was enhanced (Brady et al., 2003; Sallmyr et al., 2008).

While the regulatory influence of the ABL kinase on DSB repair has been mainly linked to DDR responses mediated by DNA-PKcs and ATM, there are also examples of modulation of DSB repair through receptor tyrosine kinases activated by their natural extracellular ligands. Over the last 15 years, evidence has accumulated that overexpression and/or mutation of the EGF receptor (EGFR) is associated with resistance to chemo- and radiotherapy (Chen and Nirodi, 2007; Dittmann et al., 2010; Toulany and Rodemann, 2010).

While EGFR is known to exert a cytoprotective action through the activation of cell survival and proliferation pathways, a large body of evidence also implicates EGFR signaling in DSB repair. In glioblastomas, brain tumors characterized by resistance to chemo- and radiotherapy, the EGFR gene is amplified in 50% of the cases and a truncated variant – EGFRvIII – is frequently over expressed. Mukherjee et al. (2009) showed that the expression of this constitutively active EGFR variant confers radioresistance through activation of PI3K-AKT signaling that enhances DSB repair.

In another study the authors showed by scoring γ-H2AX foci that activation of EGFR signaling by its natural ligand EGF enhanced the overall capacity of cells to repair DSBs (Kriegs et al., 2010). Conversely inhibition of EGFR signaling by Erlotinib or Cetuximab reduced DSB repair. The authors found that EGFR activation increased NHEJ, possibly through enhanced MAPK signaling. Another study from the same group showed a positive effect of EGFR activation on HRR (Myllynen et al., 2011). This observation is in agreement with work reported by Golding et al. (2009) who demonstrated up-regulation of both NHEJ and HRR by EGFRvIII with assays utilizing appropriate, chromosomally integrated reporter constructs.

Several reports also indicate an interference of Imatinib, Erlotinib, and other tyrosine kinase inhibitors (Chinnaiyan et al., 2005; Li et al., 2008; Choudhury et al., 2009; Zhao et al., 2011; Medova et al., 2012; Qiao et al., 2013) with HRR. These results confirm the potential impact of these signaling proteins on DSB repair and highlight their importance as targets for cancer therapy.

## DSB Repair Deficiency and Carcinogenesis

The observation that in many cancer cells the DDR is impaired emphasizes the connection between DSB repair defects and carcinogenesis and generates opportunities for cancer treatment. Indeed, various forms of cancer present with mutations or show alterations in the expression of genes encoding proteins involved in DNA metabolism (Connell et al., 2006; Klein, 2008; Miyagawa, 2008; Tennstedt et al., 2012). Furthermore, among cancers, the activities of certain repair proteins fluctuate, from complete suppression to strong up-regulation, which necessarily modulates their response to DNA damaging agents. These observations suggest that optimization of cancer therapy will benefit from in-depth analysis of the status of the DDR apparatus in each individual tumor and an adaptation of the treatment strategy to this information.

More specifically, a strong correlation between increased genomic instability, DNA repair defects, and cancer predisposition has been documented in cells isolated from individuals carrying germ line mutations in *BRCA1* or *BRCA2* genes (O’Donovan and Livingston, 2010; Roy et al., 2012). The *BRCA1* and *BRCA2* mutations increase the susceptibility to breast or ovarian cancer, and it has been estimated that the probability of developing these forms of cancer is between 30 and 80% in individuals carrying hetero- or homozygous mutations in these genes (Brody and Biesecker, 1998).

The breast cancer associated gene 1 (*BRCA1*) was identified in 1990 (Walsh and King, 2007) and was cloned a few years later (Miki et al., 1994). Biochemical and structural analyses of human BRCA1 protein revealed an N-terminal RING domain possessing E3-ubiquitin-ligase activity and a C-terminal BRCT domain, interacting with proteins phosphorylated on serine within the S-X-X-F motif. BRCA1 consists of 1863 amino acids and plays an important role in maintaining genome integrity through its functions in DNA repair (Rajagopalan et al., 2010; Roy et al., 2012) To become a functional E3-ubiquitin-ligase, the RING domain of BRCA1 acts in concert with BRCA1-associated RING domain 1 protein (BARD1), and this complex is involved in the ubiquitylation of CtIP (see above). Despite controversial studies, assigning critical functions for BRCA1 in NHEJ and nucleotide excision repair (NER), mounting evidence implicates BRCA1 as a key regulator of HRR (Jasin, 2002; Roy et al., 2012).

The direct recruitment of BRCA1 to DSBs is mediated by its interaction with Abraxas-RAP80 macro-complex, which binds to ubiquitylated proteins, particularly histones (Wang et al., 2007). Once recruited to DSBs, the main regulatory function of BRCA1 is to conscript and activate the end-resection promoting factor CtIP (Yun and Hiom, 2009). However, BRCA1 is not only implicated in HRR by its role in end-resection, but also due to its indirect interaction with BRCA2, which is mediated by the PALB2 protein (Zhang et al., 2009). Indeed, heterozygous mutations of the latter protein are also associated with predisposition to breast and pancreatic cancers (Popova et al., 2009). This interaction directly connects BRCA1 to HRR and indeed, deficiency of *BRCA1* is accompanied by abrogated formation of DSB-induced RAD51 foci and severely reduced levels of HRR.

A variety of studies with cultured cell lines has revealed that BRCA1 deficiency correlates with increased radiosensitivity to killing, which derives from the associated HRR defects (Speit and Trenz, 2004). These observations are further supported by results showing decreased survival of irradiated *BRCA1^−/−^* mouse embryonic fibroblasts exposed to IR and emphasize the central role of HRR in the maintenance of genomic integrity. Notably, more recent results suggest that inactivation of BRCA1 ubiquitin-ligase activity up-regulates protein complexes involved in DNA end-resection, causing elevated but aberrant HRR that undermines genomic instability (Drost et al., 2011; Dever et al., 2012). Along these lines, C61G mutation in the *BRCA1* gene is associated with complete loss of BRCA1 E3-ubiquitin-ligase function, and disruption of the BRCA1/BARD1 complex, which results in increased formation of RAD51 foci, and abnormal rate of HRR (Drost et al., 2011). Such results explain the observation that many sporadic BRCA1 deficient tumors develop radioresistance – possibly through enhanced aberrant HRR that triggers the function of the highly mutagenic B-NHEJ repair pathway (see above).

Although the frequency of developing breast and ovarian cancer in individuals harboring mutations in the *BRCA2* gene is lower than in individuals harboring *BRCA1* mutations, *BRCA2*-deficient patients have a 20-fold increased risk of developing prostate cancer and about 10-fold increased risk to pancreatic and other form of tumors like medulloblastomas and gliomas (Roy et al., 2012). This suggests that despite common functions in DNA repair, BRCA1 and BRCA2 also have specific functions, which explains the distinct behavior described above.

Breast cancer susceptibility gene 2 (BRCA2) is a 3418 amino acids protein, harboring a specific domain consisting of about 30 degenerative BRC repeats that are responsible for the controlled formation of RAD51 and DMC1 nucleoprotein filaments during HRR and meiosis, respectively (Thorslund and West, 2007; Roy et al., 2012). The BRC repeats exhibit subtle sequence variations allowing differential binding of RAD51 and mediating the controlled displacement of RPA from ssDNA regions and the nucleation of RAD51 monomers, which culminates with the formation of a nucleoprotein filament (West, 2003) (Figure 2). The importance of BRC repeats in BRCA2 function has been demonstrated in patients with point mutations in this domain, which develop breast and ovarian cancer with much higher frequency than patients with mutations in other regions of the gene.

Available biochemical data suggest that the main function of BRCA2 in DSB repair is to keep RAD51 in monomeric state and to deliver RAD51 monomers to the resected DNA ends. As mentioned above, the potential of BRCA2 to bind RAD51 is tightly regulated by CDK1 phosphorylation at Ser-3291, in the C-terminal TR2 motif (Esashi et al., 2005, 2007), whose abrogation results in intolerable HRR and increased genomic instability, but which might be exploited to enhance the killing potential of IR.

However, it is relevant to point out that all these well described functions in DSB repair, are not sufficient to explain initiation and progression of cancer in individuals with mutation in *BRCA* genes. Certainly, it might be speculated that BRCA1 and BRCA2 are important for tumor suppression by virtue of their function in HRR. Alternatively, it might be speculated that both proteins suppress error-prone DSB repair pathways. A strong candidate for such effects is B-NHEJ, whose involvement in DSB repair may increase when HRR is abrogated. Moreover, reports that B-NHEJ benefits from the presence of microhomology and the fact that end-resection activities like CtIP and MRN complex facilitate B-NHEJ (Xie et al., 2009; Lee-Theilen et al., 2011), support the idea that B-NHEJ may exploit failures in HRR (see above). This is especially true when limited resection of DNA ends is already accomplished, as this will prevent the recruitment of key factors of classical NHEJ. Another possibility, explaining the tumor susceptibility of BRCA-deficient patients is that the common genetic alterations (e.g., BRCA1 or BRCA2 mutations) are regularly associated with loss of wild-type p53 (Ramus et al., 1999), ATM (Tommiska et al., 2008), or CHK2 (Cao et al., 2006). These additional alterations may permit cells to bypass checkpoint controls and evade apoptosis, thereby commencing tumorigenesis.

Multiple studies link mutations in other DSB repair genes with genomic instability and cancer predisposition. Prominent among them, AT, AT like disorder (ATLD), and the NBS display mutations in genes involved in the repair of DSBs by HRR. Thus, in AT patients, ATM activity is abrogated and these individuals primarily develop lymphoid malignancies. The ATLD and NBS syndromes are associated with mutations in *MRE11* and *NBS1* genes, which together with RAD50 form the MRN complex, involved in initiation of DNA end-resection for HRR (Stracker and Petrini, 2011).

## HRR Deficiency as an Opportunity in Cancer Therapy: The Concept of Synthetic Lethality

DNA damaging agents used in cancer treatment induce a spectrum of lesions in the DNA. These lesions are recognized by a variety of cellular lesion-specific DNA repair pathways that operate to remove them from the affected DNA molecules. It is commonly accepted that DSBs are substrates for NHEJ (Lieber, 2010) and HRR (San Filippo et al., 2008). The function of these DNA repair pathways rescues malignant cells from death following exposure to radiation or chemotherapeutic drugs and compromise thus cancer treatment. It follows that inhibition of these repair processes, preferentially in malignant cells, should enhance the efficacy of cancer therapies based on killing cells by the induction of DSBs. Indeed, evidence accumulates that success in cancer treatment often results from DNA repair deficiencies in the cancer cells. Also, it has been observed that when DSB repair deficient tumors develop resistance to radiation or to DSB inducing drugs, they do so by improving their DSB repair potential (Zwet et al., 2002).

Our present understanding of DSB induction and repair allows us to postulate that combination of cytotoxic agents acting by inducing DSBs with inhibitors of DSB repair will enhance tumor cell killing – if this inhibition, or alternatively the induction of DSBs, could be somehow preferentially targeted to tumor cells. Similar arguments can be developed for other forms of DNA damage and other pathways of DNA repair.

A number of inhibitors of DNA repair have been evaluated, or are undergoing clinical trials, as potential anti-cancer chemicals. Inhibitors of PARP-1 are of particular interest in treating hereditary breast cancers occurring in patients who are carriers of *BRCA1* or *BRCA2* mutations (Bryant et al., 2005; Farmer et al., 2005). As mentioned above, BRCA2 has been established as an integral component of the HRR machinery, regulating the assembly of RAD51 filaments and facilitating strand exchange (Thorslund and West, 2007; Carreira and Kowalczykowski, 2009). Also HRR is impaired in BRCA1 deficient cells.

Poly (ADP-ribose) polymerase 1 is known to be involved in SSB repair, BER, and NER in association with XRCC1, LIG3, PNK, PCNA, and FEN1 (Frouin et al., 2003; Okano et al., 2003). PARP-1 is also involved in DSB repair (Küpper et al., 1995; Tatsumi-Miyajima et al., 1999; Rudat et al., 2001), as well as in the alternative/backup pathways of NHEJ (Wang et al., 2006; Iliakis, 2009). The combination of PARP-inhibitors with BRCA deficiency provides a sound paradigm for the power of synthetic lethality as a strategy for improving cancer treatment. Synthetic lethality emerges when the combination of non-lethal mutations in two or more genes operating in different metabolic pathways, or the chemical inhibition of their products, causes cell death.

As expected from the basic premise of synthetic lethality, PARP-1 is also effective in tumors with HRR defects deriving from genes other than BRCA – sometimes referred to as “BRCAness” (de Gonzalez et al., 2011). Thus, deficiency in *RAD51*, *RAD54*, *DSS1*, *RPA1*, *NBS1*, *ATR*, *ATM*, *CHK1*, *CHK2*, *FANCD2*, or *FANCC* genes was found to be associated with synthetic lethality to PARP inhibition (McCabe et al., 2006). These results confirm that the critical role of BRCA1 and BRCA2 in HRR is the underlying reason for the hyper-sensitivity to PARP-inhibitors of BRCA-deficient tumors. Collectively, these results indicate that the approach of synthetic lethality with PARP-1 inhibitors may prove useful for the treatment of a wide range of tumors bearing HRR deficiencies, or displaying properties of “BRCAness.”

An interesting synthetic lethal interaction has been established between RAD52 and BRCA2. Loss of RAD52 function is synthetically lethal with BRCA2 deficiency in human cancer cell lines (Feng et al., 2011; Lok et al., 2012). This suggests that BRCA2 and RAD52 provide alternative pathways for RAD51 mediated HRR in mammalian cells. RAD52 also exerts other synthetic lethal phenotypes: in chicken DT40 cells its inactivation is lethal when it occurs together with inactivation of XRCC3 (Fujimori et al., 2001). On the other hand, the viability of BRCA2-deficient DT40 cells is not compromised by deletion of RAD52; rather, an epistatic relationship between BRCA2 and RAD52 is suggested in these cells (Qing et al., 2011). Combined defects in the BRCA2 ortholog Brh2 and Rad52 generate a very subtle synthetic lethal phenotype in *U. maydis* (Kojic et al., 2008). These differences between human and other species point to the care required in the generalization of synthetic lethal interactions among species and restrict significantly the spectrum of model organisms that can be used in their study.

Poly (ADP-ribose) polymerase 1 inhibition shows synergistic interactions in combination with CHK1 inhibition and is thought to be mediated by the induction of apoptosis. CHK1 induced phosphorylation of ERK1/2 and H2AX is abolished after PARP-1 inhibition (Mitchell et al., 2010). However, the suppression of HRR by CHK1 inhibition makes this kinase an excellent target for synthetic lethality with PARP-1 inhibition also according to the rational framework outlined above.

In agreement with this expectation, Hattori et al. established a BRCA2 synthetic lethal RNAi screen, which identified CHK1 as a potential therapeutic target. Unexpectedly, though, CHK1 inhibitors failed to suppress the growth of BRCA2-deficient cells in the context of KRAS activation and TP53 inactivation found in pancreatic cancers (Hattori et al., 2011). This study extends the above outlined precautions and emphasizes that synthetic lethal interactions identified by *in vitro* screens may fail to show effectiveness in the genetic context of specific cancer forms. Evidently, the identification of synthetic lethal interactions and their exploitation in cancer therapy requires extreme care, appropriate experimentation, and model systems closely resembling the tumor whose cure is envisioned.

## Impact on Radiation Therapy of Abnormal Expression of RAD51

Cancer cell lines show fluctuations in the level of expression of genes involved in cell-cycle control and DDR. The variable expression of genes implicated in HRR, particularly overexpression of *RAD51*, is seen in many tumors and is linked to increased radio- or chemo-resistance. Moreover, a high level of RAD51 expression is observed in a variety of tumor cell lines (Richardson, 2005) and is associated with a poor outcome in the therapy of lung cancer (Qiao et al., 2005).

In addition, mammalian cells with elevated RAD51 level show genomic instability (Richardson et al., 2004), increased spontaneous recombination, and resistance to IR or to chemotherapeutic agents (Vispe et al., 1998). All these findings associate increased level of RAD51 with genomic instability and cancer development. This is surprising considering that the functions of RAD51 in HRR would predict the opposite, i.e., improved repair capacity. Indeed, it has been shown that many leukemia-related disorders, ovarian and breast carcinomas, as well as colon and rectal adenocarcinomas show, when irradiated, increased formation of RAD51 foci, which correlates with radioresistance (Raderschall et al., 2002; Klein, 2008). Moreover, the HRR deficiency of cells lacking RAD51 paralogs or BRCA1 can be completely or partially rescued by RAD51 overexpression (Schild and Wiese, 2010). Another report shows that mRNA and protein levels of RAD51, XRCC3, RAD52, and RAD54 genes are two to fivefold elevated in malignant prostate cancer cell lines (Fan et al., 2004).

Interestingly, the high RAD51 levels in these tumors are not mediated by *RAD51* gene amplification; rather overexpression is driven by aberrant oncogene related transcriptional activation. This suggests problems with the regulation of DNA metabolism in cancer cells. Major culprit for such behavior is the mutation in the tumor suppressor gene *p53*, which was found to negatively regulate RAD51 expression (Arias-Lopez et al., 2006; Hannay et al., 2007). As *p53* is the most frequently mutated gene in human cancers (Hansen et al., 2003), a RAD51-related radio- and chemo-resistance are likely consequences.

However, fluctuations in RAD51 level in tumor cells cannot be completely explained by mutations in *p53* gene. There are examples of RAD51 over expression in a functional *p53* background, suggesting a multilevel control of RAD51 expression. In these cases high RAD51 level is associated with p53-dependent expression of p21^Waf-1^, which affects the rate of BRCA1 transcription and the activation of cell-cycle checkpoint response (Walsh et al., 2011). Therefore, a strong G2 cell-cycle arrest is detected in RAD51-overexpressing cells, which occurs through p21 mediated CDK1 inactivation (Raderschall et al., 2002).

It is relevant to speculate how increased RAD51 protein levels confer radioresistance in tumor cells, especially when additional mutations are generated in DNA repair that should make them radiosensitive.

There are reports suggesting a positive correlation between HRR and high levels of RAD51. Thus, p53 deficient Chinese hamster ovary cells show elevated HRR when transfected with a vector mediating overexpression of the *RAD51* gene (Bertrand et al., 2003). Furthermore, using an I-Sce-I-based reporter system in mouse ES cells (Habrand and Le Pechoux, 2004), it was found that RAD51 over expression increases HRR. Yet, in the latter case increased HRR was associated with aberrant recombination events including crossovers, chromosome translocations, as well as multiple chromosome rearrangements and aneuploidy, suggesting frequent abrogation of HRR and possibly the engagement of B-NHEJ.

Thus, for successful HRR, RAD51 levels must be precisely regulated; otherwise chromosomal instability may ensue. The correspondence between RAD51 over expression, increased HRR, and resistance to chemotherapeutic drugs suggest the possibility for developing treatment strategies based on RAD51 down regulation – by specific inhibitors or RNAi.

## Radiosensitization by Chemotherapeutic Agents through Inhibition of HRR

As outlined above, intact or hyperactive HRR often correlates with resistance to therapy, while defects in HRR, when not associated with sensitivity to treatment, offer opportunities for synthetic lethality. It is timely, therefore, to explore means to inhibit HRR in cells proficient in this repair pathway. The potential benefit of this approach is reinforced by the observation that several compounds with antitumor activity and wide application in the clinic also inhibit HRR, and thus generate opportunities for synergistic interactions.

Radiosensitizing effects, in addition to their cytotoxic action, have been demonstrated for many chemotherapeutic drugs. Although the radiosensitizing effects of some of those drugs, e.g., 5-fluorouracil (5-FU), have been known for decades, the underlying mechanisms remain largely unclear. However, the number of radiosensitizers that are found to inhibit HRR is growing. These include nucleoside and base analogs like gemcitabine (Wachters et al., 2003), TAS-106 (Meike et al., 2011), and gimeracil (Takagi et al., 2010) as well as other antimetabolites like pentoxifylline and caffeine (Asaad et al., 2000; Böhm, 2006). Furthermore the ATR inhibitor VE-821 (Prevo et al., 2012) and the CHK1/2 inhibitor AZD7762 (Morgan et al., 2010) have been reported to inhibit HRR.

There is also a growing list of inhibitory substances and therapeutics, which are less directly linked to DNA metabolism and damage response, but which are suggested to radiosensitize tumor cells by inhibiting HRR. These include the tyrosine kinase inhibitors imatinib and erlotinib (Chinnaiyan et al., 2005; Li et al., 2008; Choudhury et al., 2009), the HDAC inhibitor PCI-24781 (Adimoolam et al., 2007), the proteasome inhibitor MG132 (Murakawa et al., 2007), and 17-AAG, an inhibitor of HSP90 (Noguchi et al., 2006). Moreover, mild hyperthermia was found to inhibit HRR and to sensitize cells to PARP-inhibitors (Krawczyk et al., 2011; Bergs et al., 2013). In addition specific inhibitors of HRR are now being identified in specialized screens, like BO2, which inhibits the RAD51 activity in strand exchange (Huang et al., 2012) and RI-1, a specific inhibitor of RAD51 that covalently binds to RAD51 and suppresses RAD51 nucleoprotein filament formation (Budke et al., 2012).

Finally, it should be pointed out that IR, but also many widely used chemotherapeutic compounds induce large amounts of base damage, or generate replication errors that require mismatch repair for correction. As a result, it is possible to significantly improve cancer treatment by developing strategies for the simultaneous inhibition during treatment of some of these repair pathways, or by exploiting genetic defects in cancer cells in these repair pathways (Kinsella, 2009).

## Conclusion

It is evident from the above outline that a wealth of information and a variety of approaches are evolving that promise to improve the outcome of radiation therapy beyond the precise and more specific targeting of the radiation dose to the tumor. These approaches are aided and accelerated by rapid advances in synthetic chemistry and in protein structure information. As a result, they are likely to mature quickly and to open a new era of opportunities in radiation therapy. Radiation oncologists are likely to benefit significantly from these developments, if they closely follow them and quickly adapt them to the requirements of treatment of human tumors by IR.

## Conflict of Interest Statement

The authors declare that the research was conducted in the absence of any commercial or financial relationships that could be construed as a potential conflict of interest.

## Appendix

**Table A1:** **Used abbreviations**.

53BP1	p53 binding protein 1
5-FU	5-fluorouracil
ATM	Ataxia telangiectasia mutated
ATR	ATM and Rad3 related
BARD1	BRCA1-associated RING domain 1 protein
BIR	Break induced replication
BLM	Bloom’s syndrome helicase
B-NHEJ	Backup non-homologous end-joining
BRCA1	Breast cancer susceptibility gene 1
BRCA2	Breast cancer susceptibility gene 2
CDKs	Cyclin dependent kinases
CHK1/2	Checkpoint kinase 1/2
CK2	Casein kinase 2
CML	Chronic myelogenous leukemia
CtIP	C-terminal binding protein 1 interacting protein
DDR	DNA damage response
dHJ	Double Holliday junction
D-loop	Displacement loop
DNA-PKcs	DNA dependent protein kinase catalytic subunit
D-NHEJ	DNA-PKcs dependent non-homologous end-joining
DSBs	Double-strand breaks
EXO1	Exonuclease 1
FEN1	Flap endonuclease 1
HDAC	Histone deacetylases
HRR	Homologous recombination repair
HU	Hydroxyurea
IR	Ionizing radiation
LIG1	DNA ligase 1
LIG3	DNA ligase 3
LIG4	DNA ligase 4
LOH	Loss of hetorozygosity
MAPK	Mitogen activated protein kinases
MRE11	Meiotic recombination 11
MRN	MRE11/RAD50/NBS1
NBS1	Nijmegen breakage syndrome 1
NHEJ	Non-homologous end-joining
PARI	PCNA-associated recombination inhibitor
PARP-1	Poly (ADP-ribose) polymerase 1
PCNA	Proliferation cell nuclear antigen
PFGE	Pulse-field gel electrophoresis
PI3K	Phosphatidyl inositol 3-OH kinases
PLK1	Polo like kinase 1
PNK	Polynucleotide kinase phosphatase
PTMs	Post-translational modifications
RPA	Replication protein A
RTEL1	Regulator of telomere elongation helicase 1
SDSA	Synthesis-dependent strand annealing
SSA	Single-strand annealing
SSBs	Single-strand breaks
ssDNA	Single-stranded DNA
SUMO	Small ubiquitin like modifiers
WRN	Werner syndrome helicase
XLF	XRCC4 like factor
XRCC1	X-ray cross complemented 1
XRCC4	X-ray cross complemented 4

## Abbreviations

All used abbreviations are indicated in Table A1 in Appendix.
